# Association of Dietary Patterns in Midlife and Cognitive Function in Later Life in US Adults Without Dementia

**DOI:** 10.1001/jamanetworkopen.2019.16641

**Published:** 2019-12-04

**Authors:** Jennifer L. Dearborn-Tomazos, Aozhou Wu, Lyn M. Steffen, Cheryl A. M. Anderson, Emily A. Hu, David Knopman, Thomas H. Mosley, Rebecca F. Gottesman

**Affiliations:** 1 Stroke Division, Department of Neurology, Harvard Medical School, Beth Israel Deaconess Medical Center, Boston, Massachusetts; 2 Department of Epidemiology, Johns Hopkins University, Baltimore, Maryland; 3 Division of Epidemiology and Community Health, University of Minnesota School of Public Health, Minneapolis; 4 Department of Family Medicine and Public Health, University of California, San Diego, San Diego; 5 Department of Neurology, Mayo Clinic, Rochester, Minnesota; 6 Division of Geriatrics, Department of Medicine, University of Mississippi Medical Center, Jackson; 7 Department of Neurology, Johns Hopkins University, Baltimore, Maryland

## Abstract

**Importance:**

The association of dietary patterns, or the combinations of different foods that people eat, with cognitive change and dementia is unclear.

**Objective:**

To examine the association of dietary patterns in midlife with cognitive function in later life in a US population without dementia.

**Design, Setting, and Participants:**

Observational cohort study with analysis of data collected from 1987 to 2017. Analysis was completed in January to February 2019. Community-dwelling black and white men and women from Washington County, Maryland; Forsyth County, North Carolina; Jackson, Mississippi; and suburban Minneapolis, Minnesota, participating in the Atherosclerosis Risk in Communities (ARIC) study were included.

**Exposures:**

Two dietary pattern scores were derived from a 66-item food frequency questionnaire using principal component analysis. A Western, or unhealthy, dietary pattern was characterized by higher consumption of meats and fried foods. A so-called prudent, or healthier, dietary pattern was characterized by higher amounts of fruits and vegetables.

**Main Outcomes and Measures:**

Results of 3 cognitive tests (Digit Symbol Substitution Test, Word Fluency Test, and Delayed Word Recall) performed at 3 points (1990-1992, 1996-1998, and 2011-2013) were standardized and combined to represent global cognitive function. The 20-year change in cognitive function was determined by tertile of diet pattern score using mixed-effect models. The risk of incident dementia was also determined by tertile of the diet pattern score.

**Results:**

A total of 13 588 participants (7588 [55.8%] women) with a mean (SD) age of 54.6 (5.7) years at baseline were included; participants in the top third of Western and prudent diet pattern scores were considered adherent to the respective diet. Cognitive scores at baseline were lower in participants with a Western diet (*z* score for tertile 3 [T3], −0.17 [95% CI, −0.20 to −0.14] vs T1, 0.17 [95% CI, 0.14-0.20]) and higher in participants with a prudent diet (*z* score for T3, −0.09 [95% CI, −0.12 to −0.06] vs T1, −0.09 [95% −0.12 to −0.06]). Estimated 20-year change in global cognitive function did not differ by dietary pattern (difference of change in *z* score for Western diet, T3 vs T1: −0.01 [95% CI, −0.05 to 0.04]; and difference of change in *z* score for prudent diet, T3 vs T1: 0.02 [95% CI, −0.02 to 0.06]). The risk of incident dementia did not differ by dietary pattern (Western hazard ratio for T3 vs T1, 1.06 [95% CI, 0.92-1.22]; prudent hazard ratio for T3 vs T1, 0.99 [95% CI, 0.88-1.12]).

**Conclusions and Relevance:**

This study found that the dietary pattern of US adults at midlife was not associated with processing speed, word fluency, memory, or incident dementia in later life.

## Key Points

**Question:**

What is the association between the Western dietary pattern in adults in midlife and cognitive decline in later life?

**Findings:**

In this cohort study of 13 588 adults without dementia at baseline, midlife dietary pattern was not associated with cognitive decline 20 years later.

**Meaning:**

The Western dietary pattern may not contribute to cognitive decline in later life.

## Teaser

This cohort study examines the association of dietary patterns in midlife with cognitive function in later life in US adults without dementia at baseline.

## Introduction

Healthy dietary patterns may protect against dementia and mild cognitive impairment.^1,2^ Prior studies demonstrate that healthy dietary patterns are associated with increased brain volumes and reduced atrophy compared with less healthy dietary patterns.^3,4^ Although the mechanism behind a healthy diet and improved brain health are not well understood, 2 plausible mechanisms include reduced vascular injury and a reduction in Alzheimer pathology.^5^ A healthy dietary pattern reduces hypertension, dysglycemia, hyperlipidemia, and chronic inflammation, which may reduce brain vascular injury.^2,6^ Second, a healthy diet may, through reduced oxidative stress, reduce the accumulation of proteins involved in Alzheimer disease.^5,7,8^

Midlife dietary pattern, compared with dietary pattern in later life, may have a stronger association with cognitive decline and dementia because chronic disease or the concern for chronic disease in later life may motivate individuals to improve their diet,^9^ making it appear that a healthy diet is associated with poor health outcomes. At least 10 prior studies examined associations of dietary patterns later in life with cognitive decline, but far fewer prospectively investigated associations for midlife dietary patterns.^9,10^ In this study, we examine the association between midlife dietary patterns and cognitive change and incident dementia over 20 years. We hypothesized that a healthy diet at midlife would be associated with less cognitive decline and a lower risk of dementia.

## Methods

### Study Population

The Atherosclerosis Risk in Communities (ARIC) study is a randomly selected and recruited observational cohort study that began in 1987 with individuals aged 45 to 64 years who were representative of the selected communities. Participants enrolled in the ARIC study were from 4 US communities (Jackson, Mississippi; Forsyth County, North Carolina; Washington County, Maryland; and suburban Minneapolis, Minnesota). A total of 15 792 participants received an initial evaluation, and these participants were re-evaluated in person every 3 years. The study is ongoing with 6 in-person visits completed to date.^11^ The current analysis includes information collected at visit 1 (1987-1989), visit 2 (1990-1992), visit 4 (1996-1998), and at visit 5 (2011-2013). All patients provided written informed consent. The study was approved by the institutional review boards at all participating institutions. The analysis presented is compliant with the Strengthening the Reporting of Observational Studies in Epidemiology (STROBE) reporting guideline.

### Exposure: Dietary Patterns

Participants completed a 66-item food frequency questionnaire at baseline (1987-1989).^12^ We condensed baseline questionnaire items into 20 food and beverage groups and derived 2 dietary pattern scores using principal components analysis with orthogonal rotation.^13^ Principal components analysis transforms possibly correlated variables (in this case, each of the 20 food groups) into a set of linearly uncorrelated variables (in this case, the 2 dietary patterns). Two distinct dietary patterns emerged with an eigenvalue greater than 2 (eTable 1 in the Supplement). The Western diet pattern explained 12% of the total variance and included higher consumption of meat, refined grains, and processed and fried foods. The so-called prudent diet pattern reflected 10% of the total variance and included higher consumption of fruits and vegetables, fish, chicken, whole grains, dairy, nuts, and alcohol. Study participants received a score for each dietary pattern. The score established how closely they adhered to a Western diet pattern or a prudent diet pattern. Scores ranged from −3.96 to 14.26 (interquartile range [IQR], −1.34 to 0.77) for the Western diet pattern and −3.56 to 10.55 (IQR, −0.98 to 0.79) for the prudent diet pattern. A higher score indicated greater adherence to each particular diet pattern.

### Outcomes

#### Cognitive Change

Cognitive testing was performed at visit 2 (1990-1992), visit 4 (1996-1998), and visit 5 (2011-2013). Three tests were used in the cognitive battery: the Delayed Word Recall (DWR)^14^ test, the Digit Symbol Substitution (DSS) test, and the Word Fluency (WF) test.^15^ A test-specific *z* score representing cognitive function at visits 4 and 5 was calculated by subtracting the baseline population mean from the participant’s raw score and dividing the difference by the baseline population standard deviation. A global *z* score representing cognitive function at visit 4 and 5 was created as the mean of the 3 test-specific *z* scores.

Cognitive change was defined as the difference in test-specific and global *z* scores at each point using random-effects linear regression models to account for the intra-individual correlation of cognitive scores.^16^

### Dementia

Dementia was adjudicated according to an established protocol that included assessments involving 3 levels of ascertainment consisting of in-person assessments, telephone interviews of participants or informants, or surveillance based on hospital discharge codes and death certificates.^17^ Dementia was adjudicated in 2011 to 2013 and 2016 to 2017. Level 1 included in-person assessment of dementia using an algorithm that incorporated information from the Clinical Dementia Rating Interview; the Mini-Mental State examination; longitudinal cognitive testing at visits 2, 4, 5, and 6; a complete neuropsychological battery at visits 5 and 6; and the Functional Activities Questionnaire.^18^ Level 2 included participants from level 1 and, in addition, 3 other categorizations: (1) participants who met predefined criteria based on completion of the Telephone Interview for Cognitive Status–modified or the Six-Item Screener, (2) deceased persons classified as having dementia, and (3) informant interviews using the AD8 dementia screening interview, as described elsewhere.^18^ Level 3 included participants in level 2 in addition to individuals with dementia identified by surveillance using prior hospital discharge codes (*International Classification of Diseases, Ninth Revision [ICD-9]* or *International Statistical Classification of Diseases and Related Health Problems, Tenth Revision [ICD-10]*) or death certificate codes for dementia.^17^ Level 3 was used for this analysis. Level 3 ascertained a dementia status (yes or no) for all participants regardless of study visit completion.

### Covariates

Covariates included demographic and lifestyle factors, clinical factors, and apolipoprotein E (*APOE*) ε4 status. Demographic factors included age, sex, race–study center, and education. Lifestyle factors included activity level, current smoking, current alcohol use, and total energy intake. Clinical factors included body mass index (calculated as weight in kilograms divided by height in meters squared), history of hypertension (yes or no, defined as use of hypertension medication, systolic blood pressure >140 mm Hg, or diastolic blood pressure >90 mm Hg at the baseline visit), diabetes (yes or no, defined as self-reported diabetes diagnosis by physicians, use of diabetes medication, or having fasting glucose level of 126 mg/dL or higher or a nonfasting glucose level of 200 mg/dL or higher at the baseline visit [to convert to millimoles per liter, multiply by 0.0555]), total cholesterol (fasting, mmol/L), history of coronary artery disease, and prevalent stroke through visit 2 defined based on self-report of stroke prior to visit 1 and adjudicated cases between visit 1 and visit 2.

### Population Attrition

There was a 15-year gap between visit 4 and visit 5, leading to attrition, largely due to death or disability. At visits 4 and 5, respectively, 73.8% and 41.4% of the original cohort remained. This dropout is likely to be informative,^19^ as we found that diet scores were associated with loss to follow-up (Table 1). To account for population attrition, we imputed the missing cognitive test results in visits 4 and 5 and missing baseline covariates using multiple imputations with chained equations.^20^ In the multiple imputations with chained equations, we incorporated the diet scores, all covariates, prior cognitive function measurements, and ancillary information about cognitive status collected prospectively for participants who did not attend visit 5. The ancillary cognitive information was collected from the Clinical Dementia Rating scale from informants of both living participants and deceased participants, the Telephone Interview for Cognitive Status for living participants, and hospitalization discharge codes and death certificates (*ICD-9* codes). We imputed the cognitive function for participants who died before visit 5 six months prior to the date of death.^21^ We conducted the primary analyses with 25 sets of imputations.

**Table 1.** Participant Characteristics by Tertile of Western Diet Score

Characteristic	No. (%)				*P* Value^a^
	Total (N = 13 588)	Tertile 1 (n = 4553)	Tertile 2 (n = 4541)	Tertile 3 (n = 4494)	
Age, mean (SD), y	54.6 (5.7)	55.1 (5.8)	54.6 (5.7)	54.0 (5.7)	<.001
Women	7588 (55.8)	3276 (72.0)	2576 (56.7)	1736 (38.6)	<.001
Black race	3191 (23.5)	1011 (22.2)	1059 (23.3)	1121 (24.9)	.008
Study center					
Forsyth County, North Carolina	3537 (26.0)	1305 (28.7)	1133 (25.0)	1099 (24.5)	<.001
Jackson, Mississippi	2844 (20.9)	899 (19.7)	967 (21.3)	978 (21.8)	
Minneapolis, Minnesota	3698 (27.2)	1256 (27.6)	1316 (29.0)	1126 (25.1)	
Washington County, Maryland	3509 (25.8)	1093 (24.0)	1125 (24.8)	1291 (28.7)	
Education					
Below high school	2856 (21.0)	748 (16.4)	908 (20.0)	1200 (26.7)	<.001
High school	5683 (41.8)	1850 (40.6)	1886 (41.5)	1947 (43.3)	
College or above	5049 (37.2)	1955 (42.9)	1747 (38.5)	1347 (30.0)	
Cigarette smoking					
Current	3297 (24.3)	777 (17.1)	1100 (24.2)	1420 (31.6)	<.001
Former	4468 (32.9)	1504 (33.1)	1495 (32.9)	1469 (32.7)	
Never	5817 (42.8)	2269 (49.9)	1944 (42.8)	1604 (35.7)	
Alcohol use					
Current	7828 (57.6)	2539 (55.8)	2660 (58.6)	2629 (58.5)	<.001
Former	2388 (17.6)	726 (15.9)	765 (16.8)	897 (20.0)	
Never	3372 (24.8)	1288 (28.3)	1116 (24.6)	968 (21.5)	
Activity level tertile: low to high					
1	5461 (40.3)	1534 (33.8)	1912 (42.2)	2015 (45.0)	<.001
2	4157 (30.7)	1468 (32.3)	1372 (30.3)	1317 (29.4)	
3	3931 (29.0)	1542 (33.9)	1245 (27.5)	1144 (25.6)	
BMI, mean (SD)	27.6 (5.3)	27.2 (5.3)	27.7 (5.3)	27.9 (5.1)	<.001
Energy, mean (SD), kcal/d	1629 (605)	1211 (375)	1535 (399)	2149 (590)	<.001
History of coronary artery disease	605 (4.5)	241 (5.3)	175 (3.9)	189 (4.2)	.002
Total cholesterol, mean (SD), mg/dL	214.7 (41.7)	217.0 (42.5)	214.7 (41.7)	212.7 (40.9)	<.001
History of hypertension	4470 (33.0)	1519 (33.5)	1520 (33.6)	1431 (31.9)	.16
History of diabetes	1437 (10.7)	488 (10.8)	501 (11.1)	448 (10.1)	.25
No. of apolipoprotein E ε4 allele					
0	9125 (69.3)	2985 (67.7)	3084 (70.0)	3056 (70.4)	.007
1	3695 (28.1)	1308 (29.7)	1194 (27.1)	1193 (27.5)	
2	339 (2.6)	115 (2.6)	130 (2.9)	94 (2.2)	
Prevalent stroke through visit 2	224 (1.6)	65 (1.4)	88 (1.9)	71 (1.6)	.15
Western diet score, mean (SD)	−0.03 (1.54)	−1.49 (0.44)	−0.28 (0.35)	1.71 (1.28)	<.001
Prudent diet score, mean (SD)	0.03 (1.41)	0.13 (1.43)	−0.10 (1.36)	0.05 (1.44)	<.001
Cohort attrition					
Missing visit 5 cognitive test	7873 (57.9)	2555 (56.1)	2612 (57.5)	2706 (60.2)	<.001
Death	3880 (28.6)	1148 (25.2)	1277 (28.1)	1455 (32.4)	<.001

Abbreviation: BMI, body mass index (calculated as weight in kilograms divided by height in meters squared).

SI conversion factor: To convert total cholesterol to mmol/L, multiply by 0.0259.

^a^ *P* value represents χ^2^ or 2-sided *t* tests as appropriate.

### Cognitive Change Modeling

In the primary analysis, we evaluated the association of the 2 dietary pattern scores by tertile with cognitive function as measured by the change in global *z* scores at visits 2, 4, and 5. Mixed-effect models were used to account for the correlation between repeated cognitive test measures over time. We defined a study time metric from visit 2 to the cognitive measurement. We used a linear spline for the time variable with a knot at visit 4 to address potential nonlinearity of cognitive change. We incorporated 2 random slopes, which corresponded to the 2 time-spline terms, and a random intercept, assuming an independent correlation structure. To measure the association between diet scores and cognitive change, we examined the interactions between exposure strata and the time-spline terms modeled as conditional likelihoods. We also included conditional likelihoods for age, sex, education, race–field center, and total energy intake for model 1 and *APOE* ε4 status, alcohol use history, smoking history, activity level, body mass index, total cholesterol, prevalent coronary heart disease, history of hypertension, diabetes, and stroke for model 2. We included the conditional likelihoods for interactions between time-splines and covariates that contributed to the slope of cognitive change for aforementioned covariates. We estimated the mean cognitive change over 20 years by dietary score tertile, using the coefficients of the 2 time-splines terms and their interactions with diet score tertile. A linear trend was tested across the dietary tertiles using the median score of each tertile modeled as a continuous variable.

We performed 2 secondary analyses. In the first, we further evaluated the association of diet scores with the *z* score from each of the 3 individual cognitive test results (DSS, DWR, and WF). In the second, we replicated the analyses using nonimputed data. In both analyses, we applied the same methods as in the primary analysis.

### Incident Dementia

We next evaluated the association of the 2 dietary pattern scores with incident dementia using Cox proportional hazard models. We adjusted for the baseline covariates age, sex, education, race–field center, and total energy intake for model 1 and *APOE* ε4 status, alcohol use history, smoking history, activity level, body mass index, total cholesterol, prevalent coronary heart disease, history of hypertension, diabetes, and stroke for model 2.

### Statistical Analysis

The analysis for this study was completed in January to February 2019. Baseline characteristics of participants were compared by tertiles of diet score using χ^2^ or analysis of variance. Two-sided *P* < .05 was considered statistically significant. Analyses were conducted using Stata statistical software version 14.2 (StataCorp).

## Results

### Participant Characteristics

A total of 15 792 adults enrolled at study baseline (1987-1989) when they were aged 45 to 64 years. Of these 15 792 participants, 6538 joined the neurocognitive visit from 2011 to 2013. Because of small numbers, and in accordance with usual ARIC practice, we excluded those who were neither white nor black (48 individuals) and the black participants in the Minnesota (22 participants) and Washington County cohorts (33 participants). We further excluded 121 participants for missing dietary data and 387 participants with implausible caloric intake, defined as less than 500 and more than 3500 total calories per day for women and less than 700 and more than 4500 total calories per day for men. We also excluded 1550 participants with incomplete cognitive data at visit 2, among whom 1348 missed visit 2, and 43 participants who were missing key covariates. The analytic population included 13 588 participants.

At the baseline visit, participants in our study had a mean (SD) age of 54.6 (5.7) years, 55.8% were women, and 37.2% had at least some college education or greater (Table 1). The average participant was overweight (mean [SD] body mass index, 27.6 [5.3]) and consumed a mean (SD) of 1629 (605) kcal/d. In all, 57.9% of participants did not complete cognitive testing at visit 5, and 28.6% of participants died between study baseline and visit 5.

### Western Diet Pattern

Adherence to the Western diet pattern was defined as participants reaching the third tertile of the Western diet pattern score. Participants with a Western diet pattern had a higher rate of study attrition (Table 1) and were less likely to be women. Participants with a Western diet pattern were more likely to be from Washington County, Maryland, or Jackson, Mississippi, compared with the other 2 sites, more likely to have less than high school education, more likely to be current smokers, and less likely to engage in physical activity. Participants with a Western diet pattern were also more likely to consume a greater number of calories but were not more likely to have hypertension and diabetes.

Cognitive scores at first measurement were lower in participants with a Western diet pattern compared with participants in the first tertile of Western diet pattern score (*z* score for tertile 3 [T3], −0.17 [95% CI, −0.20 to −0.14] vs T1, 0.17 [95% CI, 0.14-0.20]) (Table 2). The finding of lower cognitive scores in participants with a Western diet pattern was consistent after adjustments for demographic factors and caloric intake (model 1), but was not statistically significant after full adjustments for lifestyle and clinical factors (model 2) (Table 2). Twenty-year change in cognitive scores was less in participants with a Western diet pattern compared with participants in the first tertile of Western diet pattern score; however, this association did not remain after full adjustments (difference of change in *z* score for Western diet, T3 vs T1: −0.01 [95% CI, −0.05 to 0.04]) (Table 3). When examined independently, only 20-year change in the DSS *z* score was less in participants with a Western diet pattern compared with participants in the first tertile of Western diet pattern score (meaning less decline), but this association was not significant after adjustments (eTable 2 in the Supplement). Twenty-year change in the DWR or WF was not different in participants with a Western pattern compared with participants in the first tertile of Western diet pattern score (eTable 3 and eTable 4 in the Supplement).

**Table 2.** Cognitive Function at Visit 2 by Tertile of Diet Pattern

Model	Tertile 1	Tertile 2	Tertile 3	*P* Value for Trend^a^
**Western Diet Score**				
Participants, No.	4553	4541	4494	
*z* Score	0.17 (0.14 to 0.20)	0.03 (−0.00 to 0.06)	−0.17 (−0.20 to −0.14)	<.001
Model 1^b^^,^^c^	[Reference]	−0.03 (−0.06 to 0.00)	−0.04 (−0.08 to −0.00)	.04
Model 2^c^^,^^d^	[Reference]	−0.01 (−0.04 to 0.02)	−0.01 (−0.05 to 0.04)	.64
**Prudent Diet Score**				
Participants, No.	4541	4536	4511	
*z* Score	−0.09 (−0.12 to −0.06)	0.05 (0.02 to 0.08)	0.07 (0.05 to 0.10)	<.001
Model 1^b^^,^^c^	[Reference]	0.03 (0.00 to 0.06)	0.01 (−0.03 to 0.04)	.71
Model 2^b^^,^^d^	[Reference]	0.04 (0.01 to 0.07)	0.02 (−0.02 to 0.06)	.28

^a^ A linear trend was tested across the dietary tertiles using the median score of each tertile modeled as a continuous variable.

^b^ Difference in *z* score compared with the first tertile.

^c^ Model 1 adjusted for age, age squared, sex, education, race–field center, total energy intake, and income.

^d^ Model 2 adjusted for model 1 and apolipoprotein E ε4 status, alcohol use history, smoking history, activity level, body mass index, total cholesterol, prevalent coronary heart disease, and history of hypertension, diabetes, and stroke.

**Table 3.** Estimated 20-Year Change in Cognitive Function by Tertile of Diet Pattern

Change or Model	Tertile 1	Tertile 2	Tertile 3	*P* Value for Trend^a^
**Western Diet Score**				
Participants, No.	4553	4541	4494	
Change in *z* score	−1.04 (−1.09 to −0.99)	−0.97 (−1.02 to −0.92)	−0.91 (−0.98 to −0.85)	<.001
Model 1^b^^,^^c^	[Reference]	0.03 (−0.01 to 0.08)	0.04 (−0.02 to 0.10)	.17
Model 2^b^^,^^d^	[Reference]	0.03 (−0.02 to 0.07)	0.03 (−0.03 to 0.08)	.37
**Prudent Diet Score**				
Participants, No.	4541	4536	4511	
Change in *z* score	−0.92 (−0.97 to −0.87)	−0.98 (−1.04 to −0.93)	−1.02 (−1.08 to −0.96)	<.001
Model 1^b^^,^^c^	[Reference]	−0.01 (−0.06 to 0.04)	−0.03 (−0.08 to 0.02)	.24
Model 2^b^^,^^d^	[Reference]	−0.01 (−0.05 to 0.04)	−0.01 (−0.06 to 0.04)	.60

^a^ A linear trend was tested across the dietary tertiles using the median score of each tertile modeled as a continuous variable.

^b^ Difference of changes in *z* score compared with the first tertile.

^c^ Model 1 adjusted for age, age squared, sex, education, race–field center, and total calories.

^d^ Model 2 adjusted for model 1 and apolipoprotein E ε4 status, alcohol use history, smoking history, activity level, body mass index, total cholesterol, prevalent coronary heart disease, and history of hypertension, diabetes, and stroke.

Our secondary analysis using nonimputed data demonstrated the same findings for the Western diet pattern compared with the imputed data set (eTable 5 in the Supplement).

Participants with a Western diet pattern were no more likely to develop dementia 20 years later compared with participants in the first tertile of Western diet pattern score (adjusted hazard ratio for T3 vs T1, 1.06; 95% CI, 0.92-1.22 for T3 vs T1) (Table 4).

**Table 4.** Relative Risk of Incident Dementia by Diet Pattern Score^a^

Measure	Tertile 1	Tertile 2	Tertile 3	*P* Value for Trend^b^
**Western Diet Score**				
Model 1^c^				
Cases/person-years, No.^d^	865/108.2	802/106.3	739/102.5	.68
Hazard ratio (95% CI)	[Reference]	1.05 (0.94-1.16)	1.06 (0.92-1.21)	
Model 2^e^				
Cases/person-years, No.^d^	834/102.9	757/101.1	708/97.0	.88
Hazard ratio (95% CI)	[Reference]	1.01 (0.91-1.13)	1.06 (0.92-1.22)	
**Prudent Diet Score**				
Model 1^e^				
Cases/person-years, No.^d^	781/105.0	798/106.5	827/105.5	.08
Hazard ratio (95% CI)	[Reference]	0.97 (0.88-1.08)	1.04 (0.93-1.17)	
Model 2^e^				
Cases/person-years, No.^d^	750/99.7	766/101.2	783/100.1	.34
Hazard ratio (95% CI)	[Reference]	0.97 (0.87-1.07)	0.99 (0.88-1.12)	

^a^ We excluded 5 participants with invalid censoring date.

^b^ A linear trend was tested across the dietary tertiles using the median score of each tertile modeled as a continuous variable.

^c^ Model 1 adjusted for age, sex, education, race–field center, and total calories.

^d^ Person-years presented in 1000 person-years.

^e^ Model 2 further excluded 693 participants with missing covariate information and adjusted for covariates in model 1 and apolipoprotein E ε4 status, alcohol use history, smoking history, activity level, body mass index, total cholesterol, prevalent coronary heart disease, and history of hypertension, diabetes, and stroke.

### Prudent Diet Pattern

Adherence to a prudent diet pattern was defined as participants reaching the third tertile of the prudent diet pattern score (*z* score for T3, −0.09 [95% CI, −0.12 to −0.06] vs T1, −0.09 [95% −0.12 to −0.06]). Participants with a prudent diet pattern had no difference in study attrition (eTable 6 in the Supplement) and were more likely to be women. Participants with a prudent diet pattern were less likely to be from Jackson, Mississippi, and more likely to be from the other 3 study locations. Participants with a prudent diet pattern were more likely to have a college education or greater and were more likely to be never smokers and engage in physical activity. Participants with a prudent diet pattern were also more likely to consume higher calories and have diabetes but not hypertension.

Cognitive scores at first measurement were higher in participants with a prudent diet pattern compared with participants in the first tertile of prudent diet pattern score (Table 2), but this association did not remain after full adjustments. Twenty-year change in cognitive scores was greater in participants with a prudent diet pattern compared with participants in the first tertile of prudent diet pattern score; however, this association did not remain after full adjustments (difference of change in *z* score for prudent diet T3 vs T1: 0.02 [95% CI, −0.02 to 0.06]) (Table 3). When examined independently, only 20-year change in the DSS was greater in participants with a prudent diet pattern compared with participants in the first tertile of prudent diet pattern score, but this association was not significant after full adjustments (eTable 2 in the Supplement). Twenty-year change in the DWR or WF was not different in participants with a prudent diet pattern compared with participants in the first tertile of prudent diet pattern score (eTable 3 and 4 in the Supplement).

Our secondary analysis using nonimputed data demonstrated the same findings for the prudent pattern compared with the imputed data set (eTable 5 in the Supplement).

Participants with a prudent diet pattern were not more likely to develop dementia 20 years later compared with participants in the first tertile of prudent diet pattern score (adjusted hazard ratio, 0.99; 95% CI, 0.88-1.12 for T3 vs T1) (Table 4).

## Discussion

We did not find an association between dietary patterns and cognitive decline measured over 20 years. A dietary pattern high in meat and fried food intake was associated with lower cognitive test scores at baseline, but differences in demographic characteristics and health behaviors explained this finding. Similarly, a dietary pattern high in fruit and vegetable intake was associated with higher cognitive test scores at baseline, but differences in demographic characteristics and health behaviors explained this finding.

Our results stand in contrast to short-term observational studies. Several observational studies,^22,23,24,25,26^ ranging in duration from 5 to 7 years, showed modest associations between dietary patterns and cognitive health. One study^24^ followed 1410 participants over 5 years and found that adherence to a Mediterranean-type dietary pattern was associated with less decline in the Mini-Mental State examination. Another study^23^ followed more than 2200 participants for 6 years and found that the Western diet was associated with greater cognitive decline and the prudent diet was associated with less cognitive decline as measured by the Mini-Mental State examination.

A recent long-term observational study^27^ aligns with our results. The Whitehall II study^27^ measured diet in 1991 to 1993 and dementia surveillance occurred through 2017. The authors found that diet quality at midlife was not associated with incident dementia in long-term follow up. Our results confirm the findings of this study in a US population.

We suggest 3 explanations for the reported differences between short-term studies and studies with long-term follow-up.^27^ First, it may be that over time, other chronic diseases such as diabetes have a greater impact on cognition compared with diet. Our study only partially accounts for this confounding by adjusting for comorbidities at baseline. Second, participants with an unhealthy diet engage in multiple unhealthy behaviors (eg, smoking and lack of physical activity). It may be difficult to elucidate the independent outcomes associated with diet when multiple lifestyle behaviors contribute to cognitive function. Third, our study does not account for change in dietary intake or the food supply over 20 years.

Two clinical trials^28^ build on the promising observational science to examine whether dietary changes can protect against cognitive decline and dementia. One intervention^28^ tested a Mediterranean diet with olive oil or nuts as supplementation in 334 participants at high cardiovascular risk and found improved composite cognitive function compared with the control diet. A second clinical trial, the Mediterranean-Dietary Approaches to Stop Hypertension (MIND) clinical trial, is currently under way. While our study did not find an association of diet with cognitive decline, this should not undermine the potential of dietary change to affect brain health.

### Strengths and Limitations

Our study has strengths, one of which is the long duration of follow up. Another is our ability to account for study dropout due to death or loss to follow-up using criterion-standard imputation methods.^20^

There are also several limitations of our study. First, our definition of achievement of a Western or prudent diet score is based on our tertile cutoffs and may not reflect individual participant identification with the specified dietary pattern. Second, diet was measured 3 years before the first cognitive measurement. The nonconcurrent measurements are unlikely to affect the results because dietary patterns remain relatively stable up to 7 years.^29^ However, dietary intake likely changes over 20 years owing to change in the food supply and food habits. The ARIC study did not capture diet over the 20 years to test this possibility. In addition, as participants with an unhealthy diet had lower cognition at the time of first assessment, it is possible that diet exerted influence prior to our time of measurement. As diet was not associated with either cognitive trajectories or incident dementia, this is less likely to be the case. We should also note that although study dropout was accounted for, a large proportion of participants did not follow up after 20 years. Finally, as in all observational studies, we are unable to attribute causality to our observations, as the mechanisms between diet and brain health are complex, and the only way to definitively measure the relationship between dietary practices and cognition is in an experimental design in which diet is manipulated; however, long-term follow-up may be expensive.

## Conclusions

The results of this cohort study do not support the hypothesis that midlife diet significantly contributes to cognitive decline independent of demographic and behavioral factors. Our finding that participants with an unhealthy diet have lower cognitive function could be attributed to cigarette smoking, eating excess calories, or engaging in less physical activity. Our results suggest that it may be important to address all modifiable risk factors in dietary interventions, supporting the emerging body of multimodal lifestyle and behavioral research.^30^ A multimodal approach may provide greater risk reduction for cognitive aging.

## Notes

Supplement. **eTable 1.** Factor Loadings for Western and Prudent Diet Pattern Components According to Principal Components Analysis **eTable 2.** Estimated 20-Year Change in Digit Symbol Substitution (DSS) by Tertile of Diet Pattern **eTable 3.** Estimated 20-Year Change in Delayed Word Recall (DWR) by Tertile of Diet Pattern **eTable 4.** 21-Year Change in Word Fluency Test (WF) by Tertile of Diet Pattern **eTable 5.** Estimated 20-Year Change in Cognitive Function by Tertile of Diet Pattern Using Non-imputed Data **eTable 6.** Participant Characteristics by Tertile of Prudent Diet Score
