# Supplementary material for: Association of Dietary Patterns in Midlife and Cognitive Function in Later Life in US Adults Without Dementia
Source: JAMA Netw Open. 2019 Dec 4;2(12):e1916641. doi: 10.1001/jamanetworkopen.2019.16641 (PMC6902753; doi:10.1001/jamanetworkopen.2019.16641)
Supplement: Supplement. — eTable 1. Factor Loadings for Western and Prudent Diet Pattern Components According to Principal Components Analysis eTable 2. Estimated 20-Year Change in Digit Symbol Substitution (DSS) by Tertile of Diet Pattern eTable 3. Estimated 20-Year Change in Delayed Word Recall (DWR) by Tertile of Diet Pattern eTable 4. 21-Year Change in Word Fluency Test (WF) by Tertile of Diet Pattern eTable 5. Estimated 20-Year Change in Cognitive Function by Tertile of Diet Pattern Using Non-imputed Data eTable 6. Participant Characteristics by Tertile of Prudent Diet Score [file jamanetwopen-2-e1916641-s001.pdf]

Supplementary Online Content

Dearborn-Tomazos JL, Wu A, Steffen LM, et al. Association of dietary patterns in midlife and cognitive function in later life in US adults without dementia. *JAMA Netw Open*. 2019;2(12):e1916641. doi:10.1001/jamanetworkopen.2019.16641

eTable 1. Factor Loadings for Western and Prudent Diet Pattern Components According to Principal Components Analysis
eTable 2. Estimated 20-Year Change in Digit Symbol Substitution (DSS) by Tertile of Diet Pattern
eTable 3. Estimated 20-Year Change in Delayed Word Recall (DWR) by Tertile of Diet Pattern
eTable 4. 21-Year Change in Word Fluency Test (WF) by Tertile of Diet Pattern
eTable 5. Estimated 20-Year Change in Cognitive Function by Tertile of Diet Pattern Using Non-imputed Data
eTable 6. Participant Characteristics by Tertile of Prudent Diet Score

This supplementary material has been provided by the authors to give readers additional information about their work.

© 2019 Dearborn-Tomazos JL et al. *JAMA Network Open.*

eTable 1: Factor loadings for Western and Prudent Diet Pattern Components According to Principal Components Analysis

Food category	Western	Prudent
Dairy	0.023	0.334
Fat	0.189	0.178
Fruit	-0.100	0.447
Vegetable	0.057	0.450
Chicken	-0.036	0.333
Meat	0.459	0.054
Fish	-0.035	0.351
Eggs	0.314	0.038
Sweets	0.213	0.053
Nuts	0.104	0.221
French fries	0.347	-0.006
Fried foods	0.395	-0.034
Coffee	0.115	-0.023
Tea	0.065	0.069
Diet soda	-0.013	0.136
Soda	0.258	-0.149
Juice	0.146	0.015
Alcohol	0.038	-0.058
Refined grains	0.439	0.027
Whole grains	-0.079	0.338

© 2019 Dearborn-Tomazos JL et al. *JAMA Network Open.*

eTable 2: **Estimated 20-year Change in Digit Symbol Substitution (DSS) by Tertile of Diet Pattern**

	Tertile 1	Tertile 2	Tertile 3	p-trend
Western Diet Score				
N	4553	4541	4494	
Change in z-score	-0.77 (-0.80 to -0.75)	-0.73 (-0.75 to -0.70)	-0.68 (-0.70 to -0.66)	<0.001
Model 1	Reference	0.01 (-0.02 to 0.04)	0.02 (-0.02 to 0.05)	0.340
Model 2	Reference	0.00 (-0.03 to 0.03)	0.01 (-0.02 to 0.04)	0.635
Prudent Diet Score				
N	4541	4536	4511	
Change in z-score	-0.69 (-0.71 to -0.67)	-0.73 (-0.75 to -0.70)	-0.76 (-0.79 to -0.74)	<0.001
Model 1	Reference	0.01 (-0.02 to 0.04)	-0.01 (-0.04 to 0.02)	0.639
Model 2	Reference	0.01 (-0.02 to 0.04)	0.00 (-0.03 to 0.03)	0.994

Model 1: adjusted for age, sex, education, race-center, total calories
Model 2: adjusted for Model 1 and APOEe4 status, drinking history, smoking history, activity level, body mass index, total cholesterol, prevalent CHD, hypertension, diabetes, and stroke.
A linear trend was tested across the dietary tertiles using the median score of each tertile modeled as a continuous variable.

© 2019 Dearborn-Tomazos JL et al. *JAMA Network Open.*

eTable 3: Estimated 20-year Change in Delayed Word Recall (DWR) by Tertile of Diet Pattern

	Tertile 1	Tertile 2	Tertile 3	p-trend
Western Diet Score				
N	4553	4541	4494	
Change in z-score	-1.02 (-1.07 to -0.97)	-1.03 (-1.08 to -0.97)	-1.02 (-1.07 to -0.97)	0.998
Model 1	Reference	-0.02 (-0.09 to 0.05)	-0.01 (-0.08 to 0.07)	0.853
Model 2	Reference	-0.02 (-0.09 to 0.05)	-0.01 (-0.09 to 0.06)	0.804
Prudent Diet Score				
N	4541	4536	4511	
Change in z-score	-0.98 (-1.03 to -0.93)	-1.05 (-1.10 to -1.00)	-1.03 (-1.08 to -0.98)	0.268
Model 1	Reference	-0.03 (-0.10 to 0.04)	-0.01 (-0.08 to 0.06)	0.885
Model 2	Reference	-0.03 (-0.10 to 0.04)	-0.00 (-0.07 to 0.07)	0.999

Model 1: adjusted for age, sex, education, race-center, total calories
Model 2: adjusted for Model 1 and APOEe4 status, drinking history, smoking history, activity level, body mass index, total cholesterol, prevalent CHD, hypertension, diabetes, and stroke.
A linear trend was tested across the dietary tertiles using the median score of each tertile modeled as a continuous variable.

© 2019 Dearborn-Tomazos JL et al. *JAMA Network Open.*

eTable 4: 21-year Change in Word Fluency Test (WF) by Tertile of Diet Pattern

	Tertile 1	Tertile 2	Tertile 3	p-trend
Western Diet Score				
N	4553	4541	4494	
Change in z-score	-0.20 (-0.22 to -0.17)	-0.20 (-0.23 to -0.17)	-0.17 (-0.20 to -0.14)	0.219
Model 1	Reference	-0.01 (-0.05 to 0.03)	0.01 (-0.03 to 0.06)	0.434
Model 2	Reference	-0.01 (-0.05 to 0.03)	0.01 (-0.03 to 0.05)	0.559
Prudent Diet Score				
N	4541	4536	4511	
Change in z-score	-0.18 (-0.21 to -0.15)	-0.19 (-0.22 to -0.17)	-0.20 (-0.23 to -0.17)	0.335
Model 1	Reference	-0.00 (-0.04 to 0.04)	-0.01 (-0.05 to 0.03)	0.67
Model 2	Reference	0.01 (-0.03 to 0.04)	0.00 (-0.04 to 0.04)	0.985

Model 1: adjusted for age, sex, education, race-center, total calories
Model 2: adjusted for Model 1 and APOEe4 status, drinking history, smoking history, activity level, body mass index, total cholesterol, prevalent CHD, hypertension, diabetes, and stroke
A linear trend was tested across the dietary tertiles using the median score of each tertile modeled as a continuous variable.

© 2019 Dearborn-Tomazos JL et al. *JAMA Network Open.*

eTable 5: Estimated 20-year Change in Cognitive Function by Tertile of Diet Pattern Using Non-Imputed Data

	Tertile 1	Tertile 2	Tertile 3	p-trend
Western Diet Score				
N	1998	1929	1788	
Change in z-score	-0.85 (-0.88 to -0.82)	-0.82 (-0.85 to -0.79)	-0.80 (-0.83 to -0.77)	0.045
Model 1	Reference	0.01 (-0.04 to 0.05)	0.01 (-0.04 to 0.06)	0.756
Model 2	Reference	-0.00 (-0.05 to 0.04)	0.00 (-0.05 to 0.05)	0.797
Prudent Diet Score				
N	1844	1953	1918	
Change in z-score	-0.79 (-0.82 to -0.76)	-0.83 (-0.86 to -0.80)	-0.85 (-0.88 to -0.82)	0.014
Model 1	Reference	-0.00 (-0.05 to 0.04)	-0.01 (-0.05 to 0.04)	0.963
Model 2	Reference	-0.01 (-0.05 to 0.04)	-0.01 (-0.05 to 0.04)	0.818

Model 1: adjusted for age, sex, education, race-center, total calories
Model 2: adjusted for Model 1 and APOEe4 status, drinking history, smoking history, activity level, body mass index, total cholesterol, prevalent CHD, history of hypertension, diabetes, and stroke
A linear trend was tested across the dietary tertiles using the median score of each tertile modeled as a continuous variable.

© 2019 Dearborn-Tomazos JL et al. *JAMA Network Open.*

eTable 6: Participant Characteristics by Tertile of Prudent Diet Score

		Total	Tertile 1	Tertile 2	Tertile 3	p-value
N		13588	4541	4536	4511	
Age (mean, SD)		54.6 (5.7)	54.1 (5.7)	54.8 (5.7)	54.9 (5.7)	<0.001
Women		7588 (55.8%)	2316 (51.0%)	2570 (56.7%)	2702 (59.9%)	<0.001
Black		3191 (23.5%)	1316 (29.0%)	1000 (22.0%)	875 (19.4%)	<0.001
Study center	Forsyth County NC	3537 (26.0%)	1127 (24.8%)	1213 (26.7%)	1197 (26.5%)	<0.001
	Jackson MS	2844 (20.9%)	1211 (26.7%)	903 (19.9%)	730 (16.2%)	
	Minneapolis MN	3698 (27.2%)	1073 (23.6%)	1263 (27.8%)	1362 (30.2%)	
	Washington County MD	3509 (25.8%)	1130 (24.9%)	1157 (25.5%)	1222 (27.1%)	
Education	Below High school	2856 (21.0%)	1144 (25.2%)	934 (20.6%)	778 (17.2%)	<0.001
	High school	5683 (41.8%)	1976 (43.5%)	1870 (41.2%)	1837 (40.7%)	
	College or above	5049 (37.2%)	1421 (31.3%)	1732 (38.2%)	1896 (42.0%)	
Cigarette smoking	Current	3297 (24.3%)	1442 (31.8%)	1010 (22.3%)	845 (18.7%)	<0.001
	Former	4468 (32.9%)	1386 (30.5%)	1507 (33.2%)	1575 (34.9%)	
	Never	5817 (42.8%)	1709 (37.7%)	2018 (44.5%)	2090 (46.3%)	
Alcohol use	Current	7828 (57.6%)	2674 (58.9%)	2614 (57.6%)	2540 (56.3%)	0.14
	Former	2388 (17.6%)	771 (17.0%)	786 (17.3%)	831 (18.4%)	
	Never	3372 (24.8%)	1096 (24.1%)	1136 (25.0%)	1140 (25.3%)	
Activity level	1st tertiles	5461 (40.3%)	2154 (47.6%)	1769 (39.1%)	1538 (34.2%)	<0.001
	2nd tertiles	4157 (30.7%)	1359 (30.0%)	1414 (31.2%)	1384 (30.8%)	
	3rd teritles	3931 (29.0%)	1010 (22.3%)	1345 (29.7%)	1576 (35.0%)	
BMI, mean, SD (kg/m^2)		27.6 (5.3)	27.4 (5.2)	27.5 (5.2)	27.9 (5.4)	<0.001
Calories, mean, SD (kcal/day)		1629 (605)	1346 (504)	1566 (514)	1978 (613)	<0.001
History of CAD		605 (4.5%)	171 (3.8%)	193 (4.3%)	241 (5.3%)	<0.001
Total cholesterol, mean, SD (mmol/L)		5.56 (1.08)	5.56 (1.10)	5.56 (1.05)	5.57 (1.08)	0.98
History of hypertension		4470 (33.0%)	1487 (32.9%)	1512 (33.5%)	1471 (32.7%)	0.74
History of diabetes		1437 (10.7%)	394 (8.8%)	472 (10.5%)	571 (12.7%)	<0.001
# of APOE ε4 allele	0	9125 (69.3%)	3011 (68.4%)	3081 (70.1%)	3033 (69.6%)	0.21
	1	3695 (28.1%)	1284 (29.2%)	1191 (27.1%)	1220 (28.0%)	
	2	339 (2.6%)	109 (2.5%)	124 (2.8%)	106 (2.4%)	
Prevalent stroke through Visit 2		224 (1.6%)	81 (1.8%)	75 (1.7%)	68 (1.5%)	0.59
Western Diet Score (mean, SD)		-0.03 (1.54)	-0.00 (1.43)	-0.08 (1.50)	-0.00 (1.69)	0.020
Prudent Diet Score (mean, SD)		0.03 (1.41)	-1.35 (0.47)	-0.16 (0.33)	1.59 (1.14)	<0.001
Cohort Attrition	Missing visit 5 cognitive test	7873 (57.9%)	2697 (59.4%)	2583 (56.9%)	2593 (57.5%)	0.05
	Death	3880 (28.6%)	1345 (29.6%)	1263 (27.8%)	1272 (28.2%)	0.14

Values shown are No. (%) unless otherwise specified

P-value represents chi-squared or two-sided t-tests as appropriate

© 2019 Dearborn-Tomazos JL et al. *JAMA Network Open.*
